# Depressive symptoms, neuroticism, and participation in breast and cervical cancer screening: Cross‐sectional and prospective evidence from UK Biobank

**DOI:** 10.1002/pon.5272

**Published:** 2019-11-19

**Authors:** Claire L. Niedzwiedz, Kathryn A. Robb, Srinivasa Vittal Katikireddi, Jill P. Pell, Daniel J. Smith

**Affiliations:** ^1^ Institute of Health and Wellbeing University of Glasgow Glasgow UK; ^2^ MRC/CSO Social and Public Health Sciences Unit University of Glasgow Glasgow UK

**Keywords:** breast cancer, cancer, cervical cancer, depression, epidemiology, neuroticism, oncology, prevention, screening

## Abstract

**Objective:**

To assess the cross‐sectional and prospective associations between depressive symptoms, neuroticism, and participation in breast and cervical screening in the UK.

**Methods:**

Women in the UK Biobank cohort with complete data who were eligible for breast cancer screening (aged 50‐70 years, N = 143 461) and/or cervical screening (<65 years, N = 141 753) at baseline recruitment (2006‐2010) and those with follow‐up data (2014‐2019) were identified (N = 11 050 and N = 9780 for breast and cervical screening). Depressive symptoms and neuroticism were self‐reported at baseline (range 0‐12 with higher scores reflecting greater severity). Primary outcomes were reporting being up to date with breast and cervical screening. For prospective analyses, patterns of screening participation from baseline to follow‐up were identified. Logistic regression was used to analyse associations, adjusted for potential confounding factors.

**Results:**

More severe depressive symptoms were associated with reduced likelihood of breast (OR = 0.960, 95% CI: 0.950,0.970) and cervical (OR = 0.958, 95% CI: 0.950,0.966) screening participation, in cross‐sectional analyses. Higher neuroticism scores were associated with reduced cervical screening participation, but the opposite was found for breast cancer screening. Examination of individual neuroticism items revealed that anxiety and worry were associated with increased breast screening. At follow‐up, higher baseline depressive symptoms were related to decreased cervical screening (OR = 0.955, 95% CI: 0.913,0.999), but not with breast screening.

**Conclusions:**

More severe depressive symptoms may be a barrier for breast and cervical screening and could be an indicator for more proactive strategies to improve uptake.

## 1 BACKGROUND

Globally, more than two million women are diagnosed with breast or cervical cancer every year.^1^ The UK has one of the highest incidence rates of breast cancer, a leading cause of death amongst women. Cervical cancer is the 14^th^ most common cancer amongst women in the UK, although incidence has fallen over the past few decades due to effective population‐based screening programmes.^2^ The National Health Service (NHS) Cervical Screening Programme was introduced in the UK in 1988. All women who are registered with a GP are invited to attend for screening every 3 years if aged 25 to 49, every 5 years if aged 50 to 64, and only women who have recently had abnormal tests are invited if aged 65 and over. Also introduced in 1988, the NHS Breast Screening Programme currently invites all women aged between 50 and 70 for screening every 3 years.^3^

A number of social and psychological factors influence participation in breast and cervical screening. Several studies have demonstrated social inequalities in screening participation by education level,^4^ area‐level deprivation,^5^ and ethnicity.^6^ Individuals with psychiatric disorders are less likely to attend breast and cervical screening,^7^, ^8^ particularly those with severe mental illness.^9^ The presence of a high depressive symptom burden has also been associated with nonattendance at breast screening,^10^, ^11^, ^12^ but not cervical screening.^10^, ^11^

Few studies have examined the role of personality factors in cancer screening attendance.^13^, ^14^, ^15^, ^16^, ^17^ Those that have are limited by small samples and cross‐sectional designs. One important aspect of personality that could be hypothesised to impact on screening behaviour is neuroticism. Neuroticism refers to the relatively stable propensity to respond with negative emotions to threat, frustration, or loss.^18^ Neuroticism has an underlying neurobiological basis,^19^ is strongly correlated with depressive and anxiety disorders,^20^ and is associated with significant economic costs to society via mental health service usage and productivity losses.^21^ Aspects of neuroticism, such as mood instability, may decrease the likelihood of attending screening, whereas anxiety may increase screening attendance if individuals are worried about their risk of cancer.^22^ Whether neuroticism predicts screening participation independently of depressive symptoms, and whether specific aspects of neuroticism or symptoms of depression are more strongly related to screening behaviour remains to be elucidated. Greater awareness of aspects of neuroticism which affect uptake could inform public health messaging about screening programmes, as well as how health professionals communicate with individuals who may require additional support to access screening or reduce anxiety associated with it.^23^

The aim of this study was to investigate both cross‐sectional and prospective associations between depressive symptoms, neuroticism, and participation in breast and cervical screening amongst women in the UK Biobank cohort. We had four key objectives: (a) to investigate the cross‐sectional associations between depressive symptoms (overall and item‐specific questions) and participation in breast and cervical screening; (b) to examine the cross‐sectional associations between neuroticism (overall score and item‐specific questions) and participation in breast and cervical screening; (c) to examine whether baseline depressive symptoms and neuroticism scores predict future participation in breast and cervical screening; and (d) to investigate whether baseline depressive symptoms and neuroticism scores relate to longitudinal patterns of breast and cervical screening participation.

## 2 METHODS

### 2.1 Data

We used secondary data from UK Biobank (further details are found at https://www.ukbiobank.ac.uk/), which achieved a 5.5% response rate.^24^ Over 502 000 community‐dwelling individuals aged 37 to 73 years were recruited to UK Biobank during 2006 to 2010, forming the baseline sample.^25^ They attended one of 22 assessment centres distributed across England, Scotland, and Wales where data were collected on a range of topics including lifestyle, socio‐demographics, physical, and mental health. UK Biobank received ethical approval from the NHS National Research Ethics Service North West (11/NW/0382). This research has been conducted using the UK Biobank resource under Application Number 41686. All participants gave written informed consent.

We restricted the sample (Figure S1) to women aged 50 to 70 years at baseline for the breast screening analysis (N = 208 726) and women aged under 65 years at baseline for the cervical screening analysis (N = 224 805). Participants who reported that they had undergone a hysterectomy at baseline were excluded from the cervical screening analysis (N = 12 804), as they are no longer eligible for screening recall.

A repeat assessment of around 20 000 participants was carried out between August 2012 and June 2013 at the UK Biobank Co‐ordinating Centre in Cheadle, Stockport (further details in the Online Supplementary Material). A further 36 000 individuals have taken part in the UK Biobank Imaging Study at the Cheadle, Newcastle and Reading assessment centres between May 2014 and March 2019 where repeat assessment data were also collected (see https://imaging.ukbiobank.ac.uk/). We identified 13 668 and 15 107 women from the breast and cervical screening baseline samples who attended a follow‐up assessment visit. Excluding participants who were no longer eligible for screening at follow‐up on the basis of age, a total of 11 458 women were included in the breast screening prospective sample and 10 578 in the cervical screening prospective sample.

### 2.2 Outcomes

At baseline and follow‐up, participants were asked whether they had ever been for breast cancer screening (a mammogram) and whether they had ever had a cervical smear test. Those who answered “yes” were asked how many years ago their last mammogram or cervical smear test occurred. We combined these items to form baseline and follow‐up variables distinguishing those who were up to date with their breast screening (≤3 years since last screened) versus those who were not (never been for screening or >3 years since last screened). For cervical screening, “up to date with screening” was defined as those who had a cervical smear test ≤3 years ago for participants aged under 50 years and ≤5 years ago if aged 50 years and over. For the prospective analyses, we also grouped individuals into longitudinal patterns of engagement with breast and cervical screening including up to date with screening at baseline and follow‐up; up to date at baseline only; up to date at follow‐up only; or up to date at neither time point.

### 2.3 Exposure variables

Recent depressive symptoms were measured at baseline via four questions adapted from the Patient Heath Questionnaire‐9 (PHQ‐9)^26^ which were included in UK Biobank, (eg, “Over the past two weeks, how often have you felt down, depressed or hopeless?”). Participants selected either “not at all” (scored 0), “several days” (scored 1), “more than half of the days” (scored 2), or “nearly every day” (scored 3). Answers were summed to produce a scale ranging from 0 to 12, with higher scores reflecting more severe current depressive symptoms (further details in the Supporting Information). The individual items were categorised into “nearly every day” (representing probable depression) versus all others.

Neuroticism was measured at baseline via 12 questions from the brief Eysenck Personality Inventory Neuroticism Scale (EPIN‐R)^27^, ^28^ which were included in UK Biobank, (eg, “Does your mood often go up and down?”). Participants answered either “no” or “yes.” Answers were summed to produce a neuroticism score ranging from 0 to 12, where 12 represents high neuroticism.

### 2.4 Potential confounders

We included several potential baseline confounding variables: age group; ethnicity (White, Black, South Asian, Chinese, Mixed/Other); region (Scotland, England or Wales); education level; area‐level socioeconomic deprivation assessed using Townsend score^29^; self‐reported long‐standing illness or disability. Lifestyle factors including smoking (never, previous, current), body mass index (BMI), and alcohol consumption (daily or almost daily, 3‐4 times a week, once or twice a week, 1‐3 times per month, special occasions, or never) were included as potential confounding or mediating variables. Full details are provided in the Supporting Information.

### 2.5 Statistical analysis

We first descriptively examined mean neuroticism and depressive symptom scores for women who were up to date with their screening compared with those who were not. For the cross‐sectional analyses, a series of logistic regression models were calculated predicting whether participants were up to date with their breast and cervical screening (further details in the Supporting Information). We calculated logistic regression models for the two screening outcome variables including neuroticism scores, adjusted for education level, socioeconomic deprivation, ethnicity, region, and long‐standing illness or disability, followed by lifestyle factors (smoking, alcohol consumption, and BMI).

We then calculated models including the 12 individual neuroticism items (instead of the neuroticism scores as we hypothesised that there may be differences in the strength and/or direction of the associations between the different questions), adjusted for the potential confounding variables, followed by the addition of lifestyle factors.

We then tested the summed depressive symptom scores and then the four individual depressive symptom items (both adjusted for neuroticism scores). Depressive symptoms were added after adding the neuroticism items as we hypothesised that any potential causal pathway would go from neuroticism to depressive symptoms as neuroticism is a more stable trait. Predicted probabilities were calculated and graphed to help interpret the results.

We then calculated logistic regression models predicting whether participants were up to date with breast and cervical screening at follow‐up, adjusted for baseline neuroticism scores and depressive symptoms, baseline confounding variables and time between baseline and follow‐up. We then added the equivalent baseline screening variable, followed by baseline lifestyle factors.

Multinomial logistic regression models were then calculated using the longitudinal screening pattern groups as the outcome variables (with the reference group being up to date at both time points), including baseline neuroticism and depressive symptom scores, adjusted for baseline confounders and time between baseline and follow‐up. We then added the baseline lifestyle variables. All statistical models in the prospective analyses adjusted for the number of years between the baseline and follow‐up data collection (as a series of dummy variables to allow for nonlinear relationships).

We conducted complete case analyses, excluding participants with missing data for any variable. In the cross‐sectional analyses, 65 265 women in the breast cancer screening analyses and 70 248 in the cervical screening analyses had missing data and were excluded. In the prospective analyses, an additional 408 and 798 women had missing outcome data and were excluded from the breast and cervical screening analyses, respectively. Several sensitivity analyses were performed to test the robustness of the results (full details are available in the Supporting Information). Multicollinearity was checked by calculating the variance inflation factor, and potential nonlinearity was assessed but was not found to be an issue. All analyses were performed using Stata MP 15.1.

## 3 RESULTS

### 3.1 Sample description

In the cross‐sectional analyses, a total of 143 461 women were included in the breast cancer screening sample and 141 753 in the cervical screening sample (Table 1 and Table S1). Mean depressive symptom and neuroticism scores were slightly higher amongst those who were not up to date with their breast and cervical screening, compared with those who were (descriptive statistics for the individual items are found in Table S2). In the prospective analyses, a total of 11 050 women were included in the breast screening sample and 9780 in the cervical screening sample. The average number of years between baseline and follow‐up assessments was 6.3 years and 6.5 years (with a minimum of two and maximum of 12 years) for the breast and cervical screening samples, respectively. Most participants were up to date with their screening at both baseline and follow‐up (86.3% for breast screening and 83.7% for cervical screening) (Table S3).

**Table 1:** Descriptive statistics for the cross‐sectional samples

	Breast Cancer Screening		Cervical Screening	
	**N**	**%**	**N**	**%**
**Total**	**143 461**		**141 753**	
**Age group**				
<45 years	‐	‐	19 391	13.7
45‐49 years	‐	‐	25 431	17.9
50‐54 years	30 942	21.6	28 753	20.3
55‐59 years	35 614	24.8	30 991	21.9
60‐64 years	45 485	31.7	37 187	26.2
65+ years	31 420	21.9	‐	‐
**Ethnicity**				
White	138 932	96.8	135 326	95.5
Mixed or Other	1480	1.0	2121	1.5
South Asian	1275	0.9	1744	1.2
Black	1453	1.0	2115	1.5
Chinese	321	0.2	447	0.3
**Region**				
Scotland	10 690	7.5	11 034	7.8
England	126 726	88.3	124 438	87.8
Wales	6045	4.2	6281	4.4
**Education level**				
College or university degree	43 504	30.3	51 415	36.3
A levels or equivalent	16 345	11.4	18 886	13.3
GCSEs or equivalent	33 917	23.6	33 641	23.7
CSEs or equivalent	5933	4.1	8361	5.9
NVQ or equivalent	6517	4.5	6120	4.3
Other professional qualifications	9776	6.8	7386	5.2
None of the above	27 469	19.1	15 944	11.2
**Long‐standing illness**				
No	98 686	68.8	104 122	73.5
Yes	44 775	31.2	37 631	26.5
**Smoking**				
Never	83 432	58.2	85 109	60.0
Previous	48 908	34.1	43 654	30.8
Current	11 121	7.8	12 990	9.2
**Alcohol consumption**				
Daily or almost daily	25 789	18.0	23 486	16.6
Three or four times a week	30 089	21.0	31 815	22.4
Once or twice a week	36 137	25.2	38 025	26.8
One to three times a month	17 558	12.2	18 768	13.2
Special occasions only	20 698	14.4	18 628	13.1
Never	13 190	9.2	11 031	7.8

### 3.2 Cross‐sectional results

**Figure 1:**
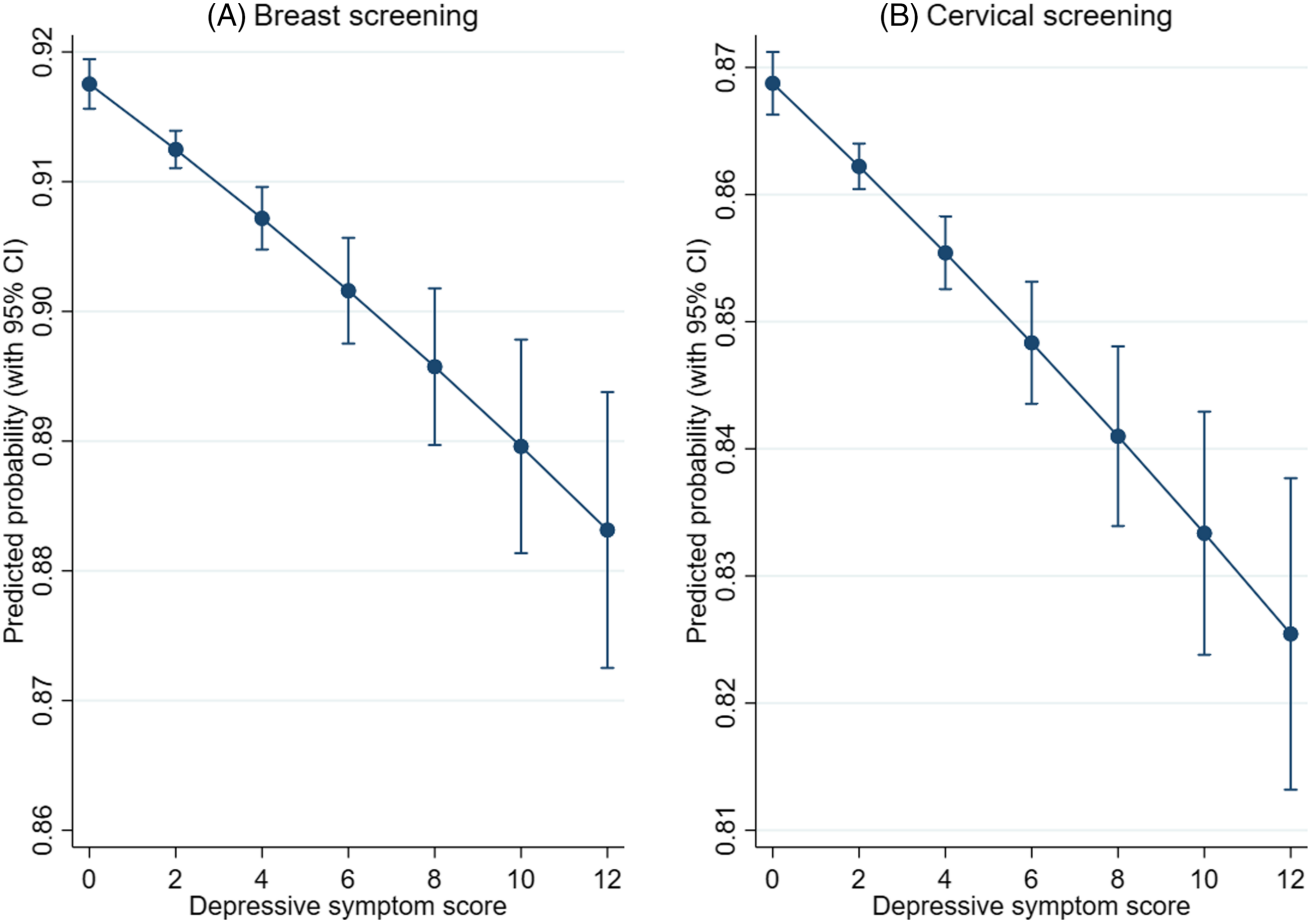
Predicted probability of screening according to depressive symptom scores

Higher neuroticism scores were associated with decreased likelihood of being up to date with cervical screening (OR = 0.978, 95% CI: 0.973,0.982) in cross‐sectional analyses (Table 2 and Table S4). This association persisted and was strengthened by the addition of depressive symptoms and lifestyle factors (smoking, alcohol consumption, and BMI). Results were less clear for breast screening. Neuroticism scores were related to increased likelihood of being up to date (OR = 1.009, 95% CI: 1.002,1.016), only after adjustment for depressive symptoms and lifestyle factors. Higher depressive symptoms were associated with decreased likelihood of being up to date with both breast (OR = 0.960, 95% CI: 0.950,0.970) and cervical screening (OR = 0.958, 95% CI: 0.950,0.966). Both associations were attenuated with the addition of lifestyle factors. The difference in the predicted probability of being up to date with screening between those with the highest and lowest depressive symptom scores was 0.034 for breast and 0.043 for cervical screening (Figure 1).

**Table 2:** Results from logistic regression models investigating the association between neuroticism, depressive symptoms, and breast (N = 143 461) and cervical (N = 141 753) cancer screening at baseline in UK Biobank

	**Breast Cancer Screening**			**Cervical Screening**		
	**Model 1**	**Model 2**	**Model 3**	**Model 1**	**Model 2**	**Model 3**
	**OR** **[95% CI]**	**OR** **[95% CI]**	**OR** **[95% CI]**	**OR** **[95% CI]**	**OR** **[95% CI]**	**OR** **[95% CI]**
**Neuroticism score**	0.995[0.989,1.001]	1.010**[1.003,1.017]	1.009*[1.002,1.016]	0.978***[0.973,0.982]	0.993*[0.988,0.999]	0.989***[0.983,0.994]
**Depressive symptoms**		0.960***[0.950,0.970]	0.966***[0.956,0.977]		0.958***[0.950,0.966]	0.972***[0.964,0.980]

CI, confidence interval; N, number of individuals.

* *P* < .05.

** *P* < .01.

*** *P* < .001.

Model 1: Neuroticism score, age group, ethnicity, education level, region, long‐standing illness, and socioeconomic deprivation.

Model 2: Model 1 + depressive symptoms.

Model 3: Model 2 + smoking + alcohol consumption + BMI.

**Figure 2:**
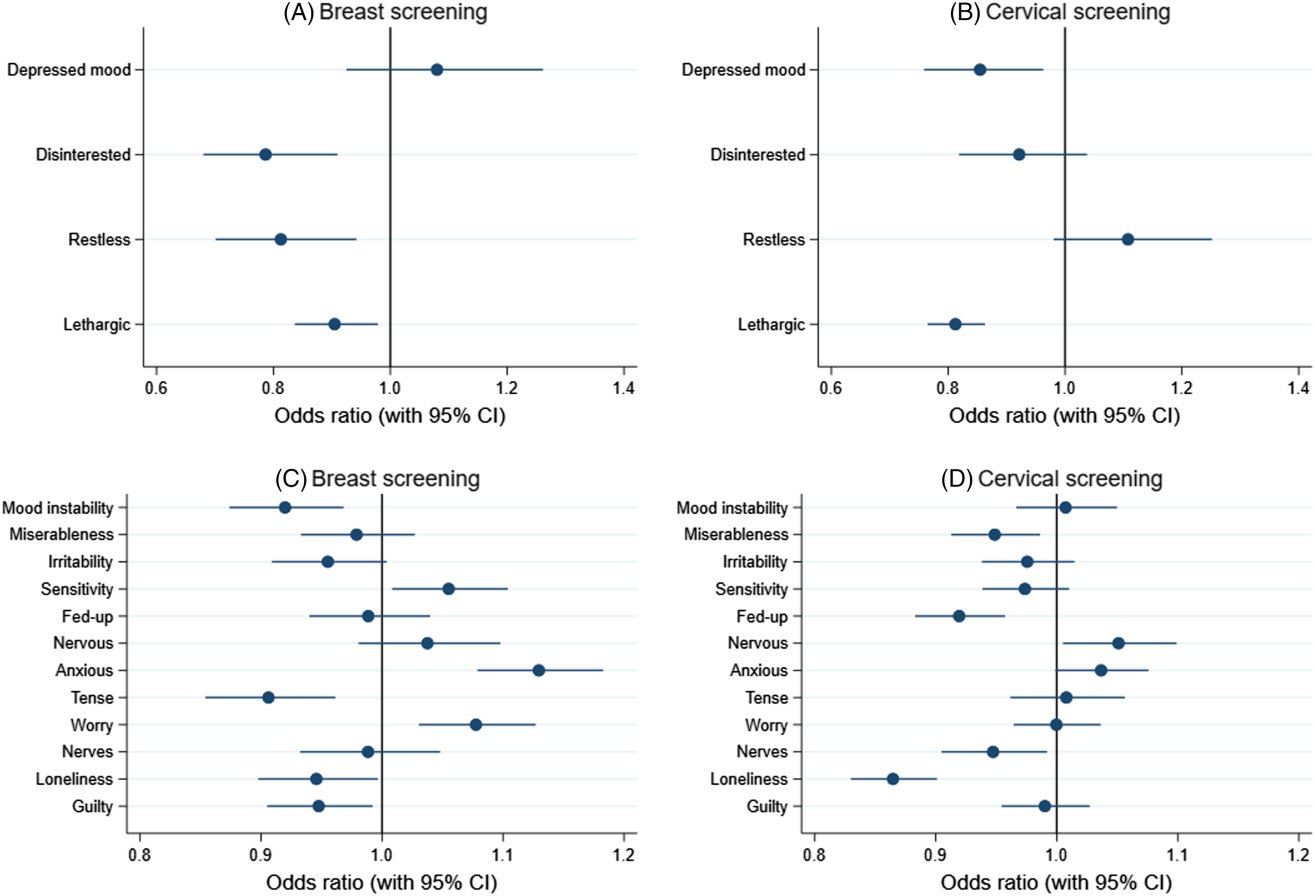
Results from cross‐sectional logistic regression models. Footnote: A. Breast screening according to depressive symptoms. B. Cervical screening according to depressive symptoms. C. Breast screening according to neuroticism items. D. Cervical screening according to neuroticism items.

Most depressive symptoms were related to reduced participation in both breast and cervical screening (Figure 2), apart from depressed mood for breast screening and restlessness for cervical screening. Feeling tired and lethargic was related to reduced participation in both breast and cervical screening.

Examination of the individual neuroticism items revealed some interesting findings (Figure 2). Being “tense or highly strung” was most strongly associated with reduced breast screening participation, whereas being “anxious or a worrier” was related to increased participation. Reporting being a nervous person was associated with being up to date with cervical screening, and loneliness was related to reduced participation in both breast and cervical screening.

### 3.3 Prospective results

Baseline depressive symptoms were not associated with breast screening at follow‐up (Table S5). A modest association was found between higher baseline depressive symptoms and reduced cervical screening at follow‐up, which persisted when adjusting for baseline cervical screening (OR = 0.955, 95% CI: 0.913,0.999). However, this was attenuated when adjusting for baseline lifestyle factors. Baseline neuroticism scores were not associated with breast or cervical screening at follow‐up. We found little evidence of associations between baseline depressive symptoms and the subsequent pattern of breast cancer screening (Tables S6 and S7).

## 4 DISCUSSION

In this large study of UK Biobank participants, we found that more severe depressive symptoms were related to reduced likelihood of being up to date with both breast and cervical screening. However, differences between those with no depressive symptoms and those with more severe symptoms were not large. Feeling tired and lethargic almost every day in the last 2 weeks was related to decreased likelihood of being up to date with both breast and cervical screening. The pattern of results was less clear for neuroticism. Overall, higher neuroticism scores were related to reduced cervical screening participation and increased breast screening participation within cross‐sectional analyses. Examination of individual items revealed that those related to anxiety and worry tended to be associated with increased breast screening, which may be driving the overall positive association. Feeling lonely was associated with reduced likelihood of being up to date with both breast and cervical screening.

Prospective analyses revealed little or no association between baseline depressive symptoms and breast cancer screening. For cervical screening, more severe depressive symptoms were associated with reduced likelihood of being up to date at follow‐up, but this was attenuated by lifestyle factors.

Our results support the findings of previous studies demonstrating that psychiatric morbidity, poor self‐reported mental health, and a higher depressive symptom burden are associated with nonparticipation in breast cancer screening.^10^, ^11^, ^12^, ^30^, ^31^ Although Vigod et al (2011) found no association between the severity of depressive symptoms and cervical screening participation in their overall sample of women aged 18 to 67 years, subgroup analyses of participants aged 40 to 67 years revealed that those with a higher depressive symptom burden were less likely to have had cervical screening in the previous 3 years. The differing pattern of associations for specific depressive symptoms between breast and cervical screening samples requires further exploration and may be partly due to different characteristics of the samples.

Very few studies have assessed the relationship between personality and cancer screening behaviour, despite numerous studies investigating how personality may influence risk of cancer^32^, ^33^, ^34^ (a link that remains highly controversial).^35^ Type A personality traits (ie, sense of time urgency, high job involvement, and competitiveness), but not hostility, were predictive of mammography use amongst a sample of postmenopausal women participating in the GAZEL Cohort Study of employees of a French national gas and electricity company.^17^ Neuroticism was also inversely associated with past, present, and future attendance at prostate cancer screening in a small sample of men in Estonia,^36^ but not with bowel cancer screening, in a study of individuals aged 60 to 75 years participating in the English Longitudinal Study of Ageing.^15^ Our study highlights the importance of examining individual neuroticism items. Items related to anxiety and worry were positively related to breast screening, and these items were likely driving the overall positive association with neuroticism scores, but they appeared less important for cervical screening. This could be because breast cancer is much more common than cervical cancer and breast cancer is also more common amongst women aged 65 to 69 years, an age group covered by UK Biobank, whereas younger people are more at risk of cervical cancer. Individuals may be more worried about getting breast cancer compared to cervical cancer, which may lead to increased screening uptake amongst individuals who have more anxious personalities.

Key strengths of our study include the large sample size provided by UK Biobank, the inclusion of both cross‐sectional and prospective analyses, and the examination of both breast and cervical screening outcomes. To our knowledge, no other study has examined the potential influence of individual neuroticism items and specific depressive symptoms, or longitudinal patterns of cancer screening behaviour.

### 4.1 Study limitations

A number of limitations are acknowledged. Although large and with a spread of individuals from different social and educational backgrounds, UK Biobank is not fully representative of the general UK population and demonstrates a “healthy volunteer” effect.^37^ Participants were also more likely to be older and live in socioeconomically advantaged areas than nonparticipants.^37^ It is also possible that individuals with more severe depressive symptoms may have been less likely to volunteer for UK Biobank, but we believe the magnitude of associations found in this study are likely to be underestimated due to any selection bias.^38^ The prospective analyses also included a relatively small number of the assessment centres. The screening outcome data were self‐reported, which we were unable to validate, and may therefore suffer from recall bias. It is possible that recall bias may be stronger amongst people with more severe depressive symptoms and higher neuroticism, with under‐ascertainment of cancer screening more likely to bias the estimate towards the null. Depressive symptoms and neuroticism were also limited by the self‐reported data collection, which may be affected by stigma.

### 4.2 Clinical implications

Cancer screening should be accessible to all in society. Ensuring that women with poor mental health are able to participate in breast and cervical screening may help to prevent future cancer morbidity. For example, if cost‐effective, women presenting to their General Practitioner with poor mental health could be reminded and offered a screening appointment if required and any anxieties could be discussed. Research could also address the acceptability of self sampling^39^ for women with psychiatric morbidity and the potential to reduce mental health inequalities in screening participation. Adequate prevention and treatment of depression could also help to improve uptake of breast and cervical screening, which may contribute to the reduction of health disparities and the prevention of multimorbidity.

## 5 CONCLUSIONS

Depressive symptoms may influence participation in cancer screening programmes. Results for neuroticism were less clear. Future research may benefit from the use of administrative cancer screening data and incorporation of objective measures of depression, such as receipt of an antidepressant prescription.

## CONFLICT OF INTEREST

J.P.P. is a member of the UK Biobank Steering Committee. The authors declare no other conflict of interest.

## Supporting information

Figure S1: Samples included in the cross‐sectional and prospective analyses for breast and cervical screening

Table S1: Descriptive statistics for the cross‐sectional samples

Table S2: Descriptive statistics for the individual neuroticism items and depressive symptoms in the cross‐sectional sample

Table S3: Descriptive statistics for the prospective samples

Table S4: Results from logistic regression models predicting whether participants were up to date with breast (N=143 461) and cervical (N=141 753) cancer screening at baseline in UK Biobank

Table S5: Results from logistic regression models predicting whether participants were up to date with breast (N=11 050) and cervical (N=9 780) cancer screening at follow‐up in UK Biobank

Table S6: Results from multinomial logistic regression models (referent category is up to date at both time points) predicting the pattern of breast cancer screening from baseline to follow‐up in UK Biobank (N= 11 050)

Table S7: Results from multinomial logistic regression models (referent category is up to date at both time points) predicting the trajectory of cervical cancer screening from baseline to followup in UK Biobank (N=9 780)

## Data Availability

UK Biobank is an open access resource. Data are available to bona fide scientists, undertaking health‐related research that is in the public good. Access procedures are described at http://www.ukbiobank.ac.uk/using-the-resource/.
