# Supplementary material for: Depressive symptoms, neuroticism, and participation in breast and cervical cancer screening: Cross‐sectional and prospective evidence from UK Biobank
Source: Psychooncology. 2019 Nov 19;29(2):381–8. doi: 10.1002/pon.5272 (PMC7002161; doi:10.1002/pon.5272)
Supplement: Supplementary file 1 — Figure S1: Samples included in the cross‐sectional and prospective analyses for breast and cervical screening [file PON-29-381-s001.pdf]

Online Supplementary Material

Figure S1: Samples included in the cross-sectional and prospective analyses for breast and cervical screening

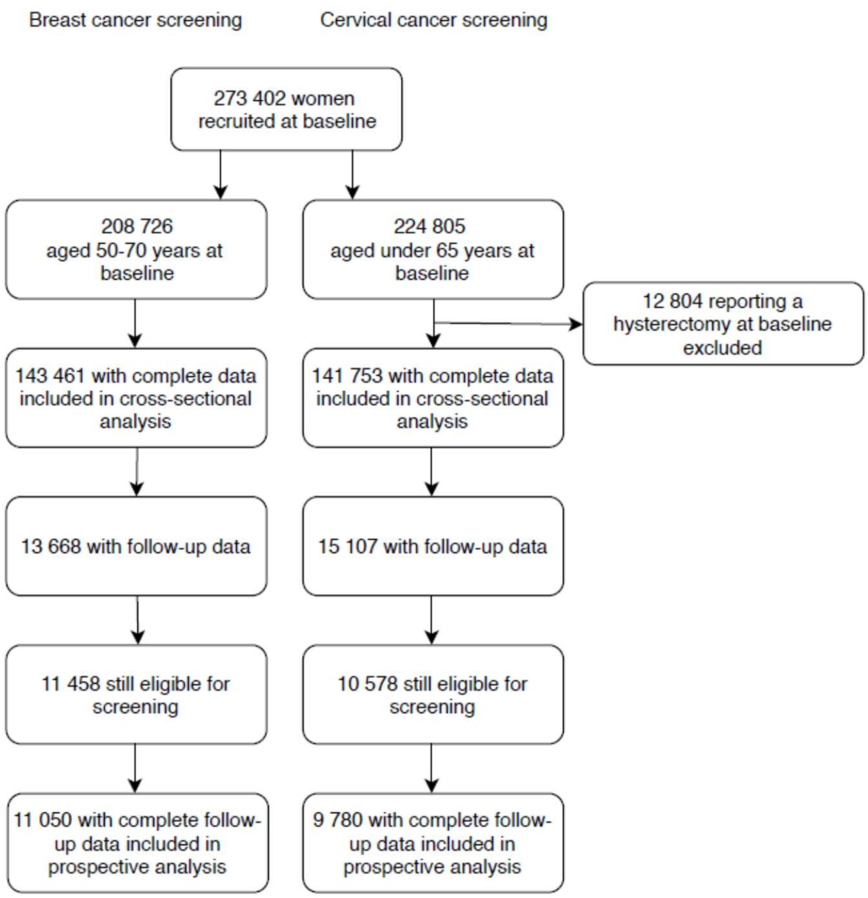

Data – Repeat Assessment Visit

A repeat assessment of 20,000 participants was carried out between August 2012 and June 2013 at the UK Biobank Co-ordinating Centre, Stockport, UK. Participants who lived within a 35 km radius of the assessment centre were invited via email or letter, with an overall response rate of 21%.[1] Participants who re-attended the assessment centre tended to have a more advantaged socioeconomic position and be in better overall health.[2]

Depressive symptoms

As detailed elsewhere[1], the questionnaire items in UK Biobank assess a) low/depressed mood ("Over the past two weeks, how often have you felt down, depressed or hopeless?"); b) anhedonia or disinterest/unenthusiasm ("Over the past two weeks, how often have you had little interest or pleasure in doing things?"); c) tenseness/restlessness ("Over the past two weeks, how often have you felt tense, fidgety or restless?") and d) tiredness/lethargy (Over the past two weeks, how often have you felt tired or had little energy?"). Participants responded "not at all", "several days", "more than half the days", or "nearly every day". Experiencing a symptom nearly every day is considered more severe than experiencing it less often. Items a), b) and d) are derived from the Patient Health Questionnaire-9 (PHQ-9), a depression screening instrument[2], and item c) is a modified version of a PHQ-9 item ("Over the last two weeks, how often have you been bothered by moving or speaking so slowly that other people could have noticed? Or the opposite – being so fidgety or restless that you have been moving around a lot more than usual?"). A total depressive symptom score from 0 to 12 was calculated from the sum of the scores on each question. A score of 12 indicates that the individual was experiencing all 4 depressive symptoms nearly every day, reflecting more severe depressive symptoms[3]. The Cronbach's alpha for the four items was 0.77. The measure has been used in previous studies using UK Biobank[1].

Potential Confounders

We included several potential baseline confounding variables including sociodemographic factors: age group (5-year age bands); ethnicity defined as white (white British, Irish or other white background), South Asian, Black (Caribbean or African), Chinese, mixed or other; and region of the assessment centre (Scotland, England or Wales). Highest education level was assessed as university or college degree, A-levels or equivalent, General Certificate of Secondary Education (GCSE) or equivalent, vocational Certificate of Secondary Education or equivalent, National Vocational Qualifications (NVQ), Higher National Diploma (HND), Higher National Certificate (HNC), other professional qualifications (e.g. nursing, teaching), or none. Area-level socioeconomic deprivation

was assessed by the Townsend score (incorporating measures of unemployment, non-car ownership, non-home ownership and household overcrowding) corresponding to the output area in which the respondent's home postcode was located. Higher scores on the Townsend score represent greater socioeconomic deprivation [6]. We also included self-reported long-standing illness, disability or infirmity (yes or no) and lifestyle factors including smoking (never, previous, current), Body Mass Index (BMI; a continuous variable calculated as weight/height^2 derived from physical measurements), and alcohol consumption (daily or almost daily, 3-4 times a week, once or twice a week, 1-3 times per month, special occasions, or never). For the prospective analyses, we collapsed the categories of several variables given the smaller sample size (details below).

Variables recoded in the prospective analysis

Ethnicity: White versus non-White

Education Level: Degree level versus non-degree level

Region: England versus not in England

Alcohol consumption: Daily (daily or almost daily), regularly (1–2 times a week or 3–4 times a week), occasionally (1–3 times a month or special occasions only), never.

Modelling strategy

Cross-sectional:

Model 1: Neuroticism score, age group, ethnicity, education level, region, long-standing illness and socioeconomic deprivation.
Rationale: Neuroticism + potential confounders

Model 2: Model 1 + depressive symptoms
Rationale: Addition of depressive symptoms as potential mediator between neuroticism and cancer screening

Model 3: Model 2 + smoking + alcohol consumption + BMI
Rationale: Addition of other lifestyle confounders or mediators.

Prospective:

Model 1: Neuroticism score, depressive symptoms, age group, ethnicity, education level, region, long-standing illness, socioeconomic deprivation and years between baseline and follow-up
Rationale: Interested in association between baseline depressive symptoms and participation in cancer screening at follow-up. Years between baseline and follow-up included as confounder.

Model 2: Model 1 + baseline cancer screening
Rationale: Baseline cancer screening added as potential confounder.

Model 3: Model 2 + smoking + alcohol consumption + BMI
Rationale: Addition of other lifestyle confounders or mediators at baseline.

Sensitivity analyses

We conducted several sensitivity analyses to test the robustness of our results. The breast cancer screening analyses were repeated excluding participants reporting that they had previously had a mastectomy (N=3 151 in the cross-sectional analyses and N=32 in prospective analyses). We did not exclude these participants at the start of the analysis as women who have had a unilateral mastectomy are still eligible for screening, but there may be a small number who have had a bilateral mastectomy who are no longer eligible. We also repeated the prospective cervical cancer screening analyses excluding participants reporting that they had a hysterectomy at follow-up (N=279). These participants were not excluded in the main analyses as it was not clear at what time point between baseline and follow-up that they had the procedure. In addition, for the breast cancer screening analysis we repeated the models excluding participants aged under 54 years as some women in this younger age group may not have been invited for screening yet. Sensitivity analyses did not affect the substantive results.

Table S1: Descriptive statistics for the cross-sectional samples

	Up to date with breast cancer screening					Up to date with cervical cancer screening				
	No		Yes		Total	No		Yes		Total
	**Mean**	**SD**	**Mean**	**SD**	-	**Mean**	**SD**	**Mean**	**SD**	-
**Neuroticism**	4.5	3.3	4.3	3.2	-	4.8	3.3	4.5	3.2	-
**Depressive symptoms**	1.9	2.3	1.5	2.0	-	2.0	2.4	1.7	2.1	-
**Socioeconomic deprivation**	-1.1	3.1	-1.6	2.9	-	-1.3	3.0	-1.5	3.0	-
**Body Mass Index**	27.2	5.5	27.2	5.0	-	27.9	5.6	26.7	5.1	-
**Age group**	**N**	**%**	**N**	**%**	**N**	**N**	**%**	**N**	**%**	**N**
<45 years	-	-	-	-	-	2 169	11.2	17 222	88.8	19 391
45-49 years	-	-	-	-	-	3 437	13.5	21 994	86.5	25 431
50-54 years	6 426	20.8	24 516	79.2	30 942	2 960	10.3	25 793	89.7	28 753
55-59 years	2 117	5.9	33 497	94.1	35 614	4 522	14.6	26 469	85.4	30 991
60-64 years	1 949	4.3	43 536	95.7	45 485	6 380	17.2	30 807	82.8	37 187
65+ years	1 973	6.3	29 447	93.7	31 420	-	-	-	-	-
Total	12 465	8.7	130 996	91.3	143 461	19 468	13.7	122 285	86.3	141 753
**Ethnicity**										
White	11 711	8.4	127 221	91.6	138 932	18 579	13.7	116 747	86.3	135 326
Mixed or Other	233	15.7	1 247	84.3	1 480	301	14.2	1 820	85.8	2 121
South Asian	240	18.8	1 035	81.2	1 275	276	15.8	1 468	84.2	1 744
Black	241	16.6	1 212	83.4	1 453	255	12.1	1 860	87.9	2 115
Chinese	40	12.5	281	87.5	321	57	12.8	390	87.2	447
Total	12 465	8.7	130 996	91.3	143 461	19 468	13.7	122 285	86.3	141 753
**Region**										
Scotland	1 119	10.5	9 571	89.5	10 690	1 322	12.0	9 712	88.0	11 034
England	10 829	8.5	115 897	91.5	126 726	17 286	13.9	107 152	86.1	124 438
Wales	517	8.6	5 528	91.4	6 045	860	13.7	5 421	86.3	6 281

Total	12 465	8.7	130 996	91.3	143 461	19 468	13.7	122 285	86.3	141 753
**Education Level**										
College or University degree	4 295	9.9	39 209	90.1	43 504	6 099	11.9	45 316	88.1	51 415
A levels or equivalent	1 573	9.6	14 772	90.4	16 345	2 417	12.8	16 469	87.2	18 886
GCSEs or equivalent	2 638	7.8	31 279	92.2	33 917	4 828	14.4	28 813	85.6	33 641
CSEs or equivalent	687	11.6	5 246	88.4	5 933	1 226	14.7	7 135	85.3	8 361
NVQ or equivalent	593	9.1	5 924	90.9	6 517	981	16.0	5 139	84.0	6 120
Other professional qualifications	723	7.4	9 053	92.6	9 776	1 173	15.9	6 213	84.1	7 386
None	1 956	7.1	25 513	92.9	27 469	2 744	17.2	13 200	82.8	15 944
Total	12 465	8.7	130 996	91.3	143 461	19 468	13.7	122 285	86.3	141 753
**Long-standing illness**										
No	8 512	8.6	90 174	91.4	98 686	12 817	12.3	91 305	87.7	104 122
Yes	3 953	8.8	40 822	91.2	44 775	6 651	17.7	30 980	82.3	37 631
Total	12 465	8.7	130 996	91.3	143 461	19 468	13.7	122 285	86.3	141 753
**Smoking**										
Never	7 099	8.5	76 333	91.5	83 432	11 517	13.5	73 592	86.5	85 109
Previous	3 878	7.9	45 030	92.1	48 908	5 801	13.3	37 853	86.7	43 654
Current	1 488	13.4	9 633	86.6	11 121	2 150	16.6	10 840	83.4	12 990
Total	12 465	8.7	130 996	91.3	143 461	19 468	13.7	122 285	86.3	141 753
**Alcohol consumption**										
Daily or almost daily	2 023	7.8	23 766	92.2	25 789	2 845	12.1	20 641	87.9	23 486
Three or four times a week	2 442	8.1	27 647	91.9	30 089	3 719	11.7	28 096	88.3	31 815
Once or twice a week	3 052	8.4	33 085	91.6	36 137	4 824	12.7	33 201	87.3	38 025
One to three times a month	1 638	9.3	15 920	90.7	17 558	2 777	14.8	15 991	85.2	18 768
Special occasions only	1 966	9.5	18 732	90.5	20 698	3 179	17.1	15 449	82.9	18 628
Never	1 344	10.2	11 846	89.8	13 190	2 124	19.3	8 907	80.7	11 031
**Total**	**12 465**	**8.7**	**130 996**	**91.3**	**143 461**	**19 468**	**13.7**	**122 285**	**86.3**	**141 753**

Table S2: Descriptive statistics for the individual neuroticism items and depressive symptoms in the cross-sectional sample

	Up to date with breast cancer screening					Up to date with cervical cancer screening				
**Neuroticism**	**No**	**No**	**Yes**	**Yes**	**Total**	**No**	**No**	**Yes**	**Yes**	**Total**
**Mood swings**	**N**	**%**	**N**	**%**	**N**	**N**	**%**	**N**	**%**	**N**
No	6,707	7.9	77,898	92.1	84,605	10,004	13.0	66,784	87.0	76,788
Yes	5,758	9.8	53,098	90.2	58,856	9,464	14.6	55,501	85.4	64,965
Total	12,465	8.7	130,996	91.3	143,461	19,468	13.7	122,285	86.3	141,753
**Miserableness**										
No	6,434	8.0	73,921	92.0	80,355	9,488	12.9	63,899	87.1	73,387
Yes	6,031	9.6	57,075	90.4	63,106	9,980	14.6	58,386	85.4	68,366
Total	12,465	8.7	130,996	91.3	143,461	19,468	13.7	122,285	86.3	141,753
**Irritability**										
No	9,231	8.3	101,991	91.7	111,222	14,031	13.5	90,090	86.5	104,121
Yes	3,234	10.0	29,005	90.0	32,239	5,437	14.4	32,195	85.6	37,632
Total	12,465	8.7	130,996	91.3	143,461	19,468	13.7	122,285	86.3	141,753
**Sensitivity / hurt feelings**										
No	4,869	8.8	50,332	91.2	55,201	6,972	12.9	47,013	87.1	53,985
Yes	7,596	8.6	80,664	91.4	88,260	12,496	14.2	75,272	85.8	87,768
Total	12,465	8.7	130,996	91.3	143,461	19,468	13.7	122,285	86.3	141,753
**Fed-up feelings**										
No	7,229	8.1	82,326	91.9	89,555	10,610	12.8	72,215	87.2	82,825
Yes	5,236	9.7	48,670	90.3	53,906	8,858	15.0	50,070	85.0	58,928
Total	12,465	8.7	130,996	91.3	143,461	19,468	13.7	122,285	86.3	141,753
**Nervous feelings**										
No	9,382	8.7	98,077	91.3	107,459	14,426	13.5	92,539	86.5	106,965
Yes	3,083	8.6	32,919	91.4	36,002	5,042	14.5	29,746	85.5	34,788
Total	12,465	8.7	130,996	91.3	143,461	19,468	13.7	122,285	86.3	141,753
**Worrier / anxious feelings**										
No	5,090	9.1	50,801	90.9	55,891	7,444	13.3	48,569	86.7	56,013
Yes	7,375	8.4	80,195	91.6	87,570	12,024	14.0	73,716	86.0	85,740

Total	12,465	8.7	130,996	91.3	143,461	19,468	13.7	122,285	86.3	141,753
**Tense / highly strung**										
No	10,030	8.5	107,802	91.5	117,832	15,568	13.5	100,059	86.5	115,627
Yes	2,435	9.5	23,194	90.5	25,629	3,900	14.9	22,226	85.1	26,126
Total	12,465	8.7	130,996	91.3	143,461	19,468	13.7	122,285	86.3	141,753
**Worry too long after embarrassment**										
No	6,118	8.8	63,119	91.2	69,237	8,910	13.4	57,756	86.6	66,666
Yes	6,347	8.6	67,877	91.4	74,224	10,558	14.1	64,529	85.9	75,087
Total	12,465	8.7	130,996	91.3	143,461	19,468	13.7	122,285	86.3	141,753
**Suffer from nerves**										
No	9,884	8.6	105,593	91.4	115,477	15,149	13.4	98,202	86.6	113,351
Yes	2,581	9.2	25,403	90.8	27,984	4,319	15.2	24,083	84.8	28,402
Total	12,465	8.7	130,996	91.3	143,461	19,468	13.7	122,285	86.3	141,753
**Loneliness, isolation**										
No	9,726	8.4	106,251	91.6	115,977	14,856	13.1	98,663	86.9	113,519
Yes	2,739	10.0	24,745	90.0	27,484	4,612	16.3	23,622	83.7	28,234
Total	12,465	8.7	130,996	91.3	143,461	19,468	13.7	122,285	86.3	141,753
**Guilty feelings**										
No	8,170	8.5	88,435	91.5	96,605	12,548	13.3	81,633	86.7	94,181
Yes	4,295	9.2	42,561	90.8	46,856	6,920	14.5	40,652	85.5	47,572
Total	12,465	8.7	130,996	91.3	143,461	19,468	13.7	122,285	86.3	141,753
**Depressive symptoms**										
**Depressed mood in last 2 weeks**										
< Every day	12,136	8.6	128,809	91.4	140,945	18,886	13.6	119,913	86.4	138,799
Nearly every day	329	13.1	2,187	86.9	2,516	582	19.7	2,372	80.3	2,954
Total	12,465	8.7	130,996	91.3	143,461	19,468	13.7	122,285	86.3	141,753
**Unenthusiasm / disinterest in last 2 weeks**										
< Every day	12,114	8.6	128,888	91.4	141,002	18,936	13.6	119,968	86.4	138,904
Nearly every day	351	14.3	2,108	85.7	2,459	532	18.7	2,317	81.3	2,849
Total	12,465	8.7	130,996	91.3	143,461	19,468	13.7	122,285	86.3	141,753
**Tenseness / restlessness in last 2 weeks**										

< Every day	12,133	8.6	129,013	91.4	141,146	18,986	13.7	120,060	86.3	139,046
Nearly every day	332	14.3	1,983	85.7	2,315	482	17.8	2,225	82.2	2,707
Total	12,465	8.7	130,996	91.3	143,461	19,468	13.7	122,285	86.3	141,753
**Tiredness / lethargy in last 2 weeks**										
< Every day	11,411	8.5	123,311	91.5	134,722	17,672	13.4	114,259	86.6	131,931
Nearly every day	1,054	12.1	7,685	87.9	8,739	1,796	18.3	8,026	81.7	9,822
Total	12,465	8.7	130,996	91.3	143,461	19,468	13.7	122,285	86.3	141,753

Table S3: Descriptive statistics for the prospective samples

	Breast cancer screening trajectory				Cervical cancer screening trajectory			
**Neuroticism**	**Mean**	**SD**	**N**	**%**	**Mean**	**SD**	**N**	**%**
Up to date at both time points	4.1	3.2	9 541	86.3	4.4	3.2	8 181	83.7
Up to date at baseline only	4.1	3.1	542	4.9	4.3	3.3	486	5.0
Up to date at follow-up only	4.4	3.2	783	7.1	4.6	3.3	282	2.9
Up to date at neither time points	3.7	3.1	184	1.7	4.6	3.2	831	8.5
Total	4.1	3.2	11 050	100.0	4.4	3.2	9 780	100.0
**Depressive symptoms**								
Up to date at both time points	1.3	1.8	9 541	86.3	1.6	1.9	8 181	83.7
Up to date at baseline only	1.4	1.8	542	4.9	1.8	2.2	486	5.0
Up to date at follow-up only	1.7	2.1	783	7.1	1.8	2.1	282	2.9
Up to date at neither time points	1.3	2.0	184	1.7	1.9	2.3	831	8.5
Total	1.4	1.8	11 050	100.0	1.6	2.0	9 780	100.0

N=number of individuals; SD=standard deviation

Table S4: Results from logistic regression models predicting whether participants were up to date with breast (N=143 461) and cervical (N=141 753) cancer screening at baseline in UK Biobank

	**Breast cancer screening**			**Cervical cancer screening**		
	**Model 1**	**Model 2**	**Model 3**	**Model 1**	**Model 2**	**Model 3**
	**OR [95% CI]**	**OR [95% CI]**	**OR [95% CI]**	**OR [95% CI]**	**OR [95% CI]**	**OR [95% CI]**
**Neuroticism score**	0.995 [0.989,1.001]	1.010** [1.003,1.017]	1.009* [1.002,1.016]	0.978*** [0.973,0.982]	0.993* [0.988,0.999]	0.989*** [0.983,0.994]
**Depressive symptoms**		0.960*** [0.950,0.970]	0.966*** [0.956,0.977]		0.958*** [0.950,0.966]	0.972*** [0.964,0.980]
**Ethnicity (ref=White)**						
Mixed or Other	0.653*** [0.563,0.758]	0.668*** [0.576,0.775]	0.696*** [0.600,0.808]	0.934 [0.825,1.058]	0.957 [0.844,1.084]	1.032 [0.910,1.170]
South Asian	0.500*** [0.431,0.581]	0.521*** [0.449,0.605]	0.550*** [0.473,0.641]	0.815** [0.715,0.929]	0.848* [0.744,0.967]	0.983 [0.860,1.123]
Black	0.713*** [0.615,0.827]	0.732*** [0.631,0.849]	0.741*** [0.638,0.860]	1.188* [1.039,1.359]	1.216** [1.063,1.391]	1.415*** [1.236,1.621]
Chinese	0.798 [0.566,1.125]	0.814 [0.578,1.148]	0.866 [0.613,1.223]	0.951 [0.719,1.259]	0.972 [0.734,1.286]	1.030 [0.778,1.365]
**Education Level (ref=Degree)**						
A-Levels	0.997 [0.937,1.062]	0.997 [0.937,1.062]	1.012 [0.951,1.078]	0.929** [0.883,0.977]	0.930** [0.884,0.978]	0.964 [0.916,1.014]
O-Levels	1.119*** [1.061,1.179]	1.119*** [1.062,1.180]	1.148*** [1.089,1.211]	0.847*** [0.813,0.883]	0.848*** [0.814,0.884]	0.900*** [0.863,0.938]
Certificate of Secondary Education	0.963 [0.881,1.053]	0.966 [0.884,1.056]	0.998 [0.912,1.091]	0.797*** [0.745,0.852]	0.801*** [0.749,0.857]	0.868*** [0.812,0.929]
National Vocational Qualification	1.051 [0.957,1.154]	1.060 [0.965,1.164]	1.112* [1.012,1.222]	0.766*** [0.711,0.825]	0.773*** [0.718,0.833]	0.842*** [0.781,0.907]
Other	1.060 [0.974,1.154]	1.063 [0.976,1.157]	1.091* [1.002,1.187]	0.784*** [0.732,0.840]	0.788*** [0.736,0.844]	0.838*** [0.782,0.898]
None	1.038 [0.978,1.103]	1.047 [0.986,1.112]	1.115*** [1.048,1.185]	0.793*** [0.753,0.835]	0.802*** [0.762,0.845]	0.892*** [0.847,0.941]
**Region (ref=England)**						

Scotland	0.815*** [0.762,0.872]	0.815*** [0.762,0.872]	0.819*** [0.766,0.876]	1.159*** [1.091,1.231]	1.158*** [1.091,1.231]	1.171*** [1.102,1.244]
Wales	0.981 [0.892,1.078]	0.987 [0.897,1.085]	0.992 [0.902,1.091]	1.022 [0.948,1.100]	1.028 [0.954,1.107]	1.060 [0.983,1.142]
**Long-standing illness (ref=No)**						
Yes	0.925*** [0.887,0.964]	0.950* [0.911,0.991]	0.974 [0.933,1.017]	0.700*** [0.678,0.724]	0.721*** [0.697,0.746]	0.787*** [0.761,0.815]
**Socioeconomic deprivation**	0.955*** [0.949,0.961]	0.957*** [0.951,0.963]	0.964*** [0.958,0.971]	0.984*** [0.979,0.989]	0.987*** [0.981,0.992]	0.997 [0.991,1.002]
**Smoking (ref=Never)**						
Current			0.965 [0.924,1.007]			1.063*** [1.026,1.100]
Previous			0.685*** [0.643,0.731]			0.827*** [0.785,0.871]
**Alcohol consumption (ref=Daily)**						
3-4 times/week			1.029 [0.966,1.096]			1.012 [0.960,1.066]
1-2 times/week			0.985 [0.927,1.047]			0.962 [0.914,1.011]
1-3 times/month			0.906** [0.843,0.973]			0.847*** [0.799,0.897]
Special occasions only			0.856*** [0.798,0.917]			0.773*** [0.730,0.818]
Never			0.792*** [0.732,0.856]			0.679*** [0.636,0.724]
**Body Mass Index**			1.002 [0.998,1.006]			0.974*** [0.971,0.977]

CI=Confidence interval; N=number of individuals. * $p < 0.05$, ** $p < 0.01$, *** $p < 0.001$.

Model 1: Neuroticism score, age group, ethnicity, education level, region, long-standing illness and socioeconomic deprivation

Model 2: Model 1 + depressive symptoms

Model 3: Model 2 + smoking + alcohol consumption + BMI

Table S5: Results from logistic regression models predicting whether participants were up to date with breast (N=11 050) and cervical (N=9 780) cancer screening at follow-up in UK Biobank

	**Breast cancer screening**			**Cervical cancer screening**		
	**Model 1**	**Model 2**	**Model 3**	**Model 1**	**Model 2**	**Model 3**
	**OR [95% CI]**	**OR [95% CI]**	**OR [95% CI]**	**OR [95% CI]**	**OR [95% CI]**	**OR [95% CI]**
**Neuroticism**	1.018 [0.989,1.048]	1.017 [0.987,1.047]	1.016 [0.987,1.047]	1.016 [0.993,1.038]	1.020 [0.991,1.050]	1.017 [0.988,1.047]
**Depressive symptoms**	0.988 [0.940,1.039]	0.995 [0.946,1.047]	1.001 [0.951,1.053]	0.942*** [0.911,0.975]	0.955* [0.913,0.999]	0.963 [0.920,1.007]
**Up to date with screening at baseline (ref=No)**						
Yes		4.765*** [3.901,5.822]	4.742*** [3.881,5.795]		55.531*** [46.696,66.039]	54.632*** [45.918,65.001]
**Ethnicity (ref=White)**						
Non-white	0.413*** [0.273,0.624]	0.433*** [0.283,0.664]	0.463*** [0.301,0.713]	0.698* [0.508,0.960]	0.580* [0.380,0.886]	0.605* [0.394,0.929]
**Education Level (ref=Degree)**						
Non-degree	1.232** [1.056,1.436]	1.230** [1.053,1.438]	1.241** [1.060,1.452]	0.919 [0.816,1.035]	0.928 [0.795,1.084]	0.957 [0.818,1.120]
**Region (ref=England)**						
Outside England	0.717 [0.477,1.077]	0.755 [0.495,1.151]	0.750 [0.492,1.144]	1.203 [0.820,1.765]	0.824 [0.520,1.305]	0.821 [0.519,1.300]
**Long-standing illness (ref=No)**						
Yes	1.033 [0.863,1.235]	1.029 [0.858,1.235]	1.023 [0.851,1.230]	0.663*** [0.580,0.757]	0.712*** [0.595,0.851]	0.753** [0.627,0.904]
**Socioeconomic**	0.944***	0.948***	0.951***	0.994	1.014	1.019

**deprivation**	[0.918,0.971]	[0.921,0.975]	[0.924,0.979]	[0.973,1.017]	[0.984,1.044]	[0.989,1.050]
**Smoking (ref=Never)**						
Current			1.062 [0.892,1.265]			0.972 [0.816,1.159]
Previous			0.674* [0.491,0.923]			0.953 [0.684,1.327]
**Alcohol consumption (ref=Daily)**						
Regularly			1.084 [0.878,1.340]			0.870 [0.691,1.095]
Occasionally			0.920 [0.722,1.173]			0.866 [0.668,1.123]
Never			0.692* [0.494,0.970]			0.738 [0.508,1.071]
**Body Mass Index**			1.015 [0.998,1.033]			0.978** [0.963,0.993]

CI=Confidence interval; N=number of individuals. * $p < 0.05$, ** $p < 0.01$, *** $p < 0.001$.
Model 1: Neuroticism score, depressive symptoms, age group, ethnicity, education level, region, long-standing illness, socioeconomic deprivation and years between baseline and follow-up
Model 2: Model 1 + baseline cancer screening
Model 3: Model 2 + smoking + alcohol consumption + BMI

Table S6: Results from multinomial logistic regression models (referent category is up to date at both time points) predicting the pattern of breast cancer screening from baseline to follow-up in UK Biobank (N= 11 050)

	Up to date at baseline only		Up to date at follow-up only		Up to date at neither time point	
	**Model 1**	**Model 2**	**Model 1**	**Model 2**	**Model 1**	**Model 2**
	**RRR [95% CI]**	**RRR [95% CI]**	**RRR [95% CI]**	**RRR [95% CI]**	**RRR [95% CI]**	**RRR [95% CI]**
**Neuroticism score**	0.988 [0.956,1.021]	0.989 [0.957,1.023]	0.996 [0.968,1.025]	0.998 [0.970,1.027]	0.963 [0.909,1.019]	0.961 [0.907,1.017]
**Depressive symptoms**	1.023 [0.967,1.083]	1.016 [0.959,1.076]	1.049* [1.002,1.098]	1.047 [1.000,1.096]	0.998 [0.901,1.105]	0.999 [0.902,1.106]
**Age group (ref=50-54 years)**						
55-59 years	1.035 [0.834,1.285]	1.039 [0.837,1.290]	0.144*** [0.118,0.177]	0.146*** [0.119,0.179]	0.590** [0.415,0.840]	0.591** [0.415,0.843]
60-64 years	1.101 [0.860,1.408]	1.112 [0.868,1.423]	0.093*** [0.070,0.122]	0.094*** [0.071,0.124]	0.537** [0.357,0.808]	0.545** [0.361,0.822]
65+ years	2.640*** [1.719,4.053]	2.673*** [1.739,4.108]	0.168*** [0.095,0.299]	0.171*** [0.096,0.303]	1.420 [0.753,2.679]	1.453 [0.768,2.748]
**Ethnicity (ref=White)**						
Non-white	2.148** [1.305,3.537]	2.030** [1.227,3.359]	1.184 [0.720,1.949]	1.184 [0.717,1.955]	3.456*** [1.756,6.802]	3.119** [1.569,6.196]
**Education level (ref=Degree)**						
Non-degree	0.860 [0.720,1.026]	0.853 [0.714,1.021]	1.017 [0.872,1.187]	1.019 [0.872,1.191]	0.691* [0.512,0.931]	0.683* [0.505,0.923]
**Region (ref=England)**						
Outside of England	1.126 [0.699,1.815]	1.141 [0.708,1.839]	1.063 [0.681,1.661]	1.067 [0.683,1.668]	2.812** [1.331,5.942]	2.798** [1.321,5.927]
**Long-standing illness (ref=No)**	1.126 [0.699,1.815]	1.141 [0.708,1.839]	1.063 [0.681,1.661]	1.067 [0.683,1.668]	2.812** [1.331,5.942]	2.798** [1.321,5.927]
Yes	1.018 [0.831,1.248]	1.014 [0.824,1.246]	1.021 [0.852,1.224]	1.014 [0.843,1.218]	0.827 [0.574,1.192]	0.856 [0.592,1.238]
**Socioeconomic deprivation**	1.067*** [1.033,1.101]	1.063*** [1.029,1.098]	1.033* [1.004,1.063]	1.034* [1.005,1.064]	1.049 [0.993,1.109]	1.046 [0.989,1.105]

**Smoking (ref=Never)**						
Previous		0.873 [0.716,1.065]		0.848 [0.712,1.011]		1.080 [0.777,1.501]
Current		1.521* [1.075,2.153]		1.062 [0.761,1.482]		1.403 [0.740,2.660]
**Alcohol consumption (ref=Daily)**						
Regularly		0.895 [0.706,1.133]		1.006 [0.811,1.249]		1.017 [0.668,1.547]
Occasionally		0.992 [0.754,1.305]		1.025 [0.799,1.314]		1.489 [0.933,2.374]
Never		1.328 [0.906,1.946]		0.969 [0.660,1.423]		1.833 [0.972,3.457]
**Body Mass Index**		0.995 [0.976,1.014]		1.005 [0.989,1.021]		0.949** [0.916,0.984]

CI=Confidence interval; RRR=Relative Risk Ratio. * $p < 0.05$, ** $p < 0.01$, *** $p < 0.001$.
Model 1: Neuroticism score, depressive symptoms, age group, ethnicity, education level, region, long-standing illness, socioeconomic deprivation and years between baseline and follow-up
Model 2: Model 1 + smoking + alcohol consumption + BMI

Table S7: Results from multinomial logistic regression models (referent category is up to date at both time points) predicting the trajectory of cervical cancer screening from baseline to follow-up in UK Biobank (N=9 780)

	Up to date at baseline only		Up to date at follow-up only		Up to date at neither time point	
	**Model 1**	**Model 2**	**Model 1**	**Model 2**	**Model 1**	**Model 2**
	**RRR [95% CI]**	**RRR [95% CI]**	**RRR [95% CI]**	**RRR [95% CI]**	**RRR [95% CI]**	**RRR [95% CI]**
**Neuroticism**	0.973 [0.940,1.008]	0.976 [0.943,1.011]	0.994 [0.951,1.040]	0.998 [0.954,1.044]	0.991 [0.965,1.018]	0.998 [0.971,1.025]
**Depressive symptoms**	1.060* [1.005,1.118]	1.051 [0.996,1.109]	1.049 [0.980,1.124]	1.036 [0.966,1.111]	1.065** [1.022,1.109]	1.046* [1.003,1.090]
**Age group (ref=40-44 years)**						
45-49 years	0.721* [0.540,0.962]	0.717* [0.537,0.957]	1.182 [0.889,1.571]	1.172 [0.881,1.559]	1.270 [0.981,1.644]	1.276 [0.984,1.653]
50-54 years	1.230 [0.954,1.587]	1.201 [0.930,1.551]	0.226*** [0.147,0.347]	0.219*** [0.142,0.337]	1.624*** [1.272,2.073]	1.608*** [1.258,2.056]
55-59 years	1.008 [0.738,1.377]	0.978 [0.715,1.339]	0.384*** [0.250,0.589]	0.372*** [0.242,0.572]	2.228*** [1.723,2.881]	2.252*** [1.739,2.917]
>60 years	0.967 [0.474,1.973]	0.932 [0.456,1.904]	0.374* [0.143,0.976]	0.354* [0.135,0.927]	3.029*** [2.031,4.519]	2.967*** [1.984,4.435]
**Ethnicity (ref=White)**						
Non-white	1.623* [1.028,2.560]	1.606* [1.012,2.551]	0.657 [0.304,1.423]	0.655 [0.300,1.428]	1.280 [0.851,1.925]	1.173 [0.776,1.773]
**Education level (ref=Degree)**						
Non-degree	0.991 [0.823,1.193]	0.958 [0.794,1.155]	0.863 [0.678,1.098]	0.830 [0.650,1.059]	1.142 [0.986,1.322]	1.072 [0.924,1.244]
**Region (ref=England)**						
Outside of England	0.948 [0.569,1.577]	0.933 [0.561,1.552]	0.276** [0.107,0.714]	0.271** [0.105,0.701]	0.673 [0.388,1.167]	0.664 [0.383,1.154]
**Long-standing illness (ref=No)**						
Yes	1.288* [1.035,1.603]	1.217 [0.974,1.519]	0.937 [0.687,1.278]	0.880 [0.642,1.206]	1.636*** [1.394,1.921]	1.474*** [1.251,1.738]
**Socioeconomic deprivation**	0.985	0.979	1.030	1.024	1.020	1.010

	[0.951,1.020]	[0.944,1.015]	[0.987,1.076]	[0.979,1.070]	[0.993,1.048]	[0.983,1.039]
**Smoking (ref=Never)**						
Previous		1.107 [0.900,1.363]		1.010 [0.765,1.334]		0.876 [0.740,1.038]
Current		1.044 [0.701,1.556]		0.972 [0.587,1.610]		1.035 [0.758,1.412]
**Alcohol consumption (ref=Daily)**						
Regularly		1.165 [0.885,1.533]		1.008 [0.709,1.433]		1.123 [0.896,1.409]
Occasionally		1.035 [0.753,1.421]		1.022 [0.686,1.522]		1.517*** [1.187,1.939]
Never		1.250 [0.795,1.964]		0.905 [0.472,1.733]		1.465* [1.035,2.073]
**Body Mass Index**		1.032*** [1.014,1.051]		1.043*** [1.019,1.067]		1.043*** [1.029,1.057]

CI=Confidence interval; RRR=Relative Risk Ratio. * $p < 0.05$, ** $p < 0.01$, *** $p < 0.001$.
Model 1: Neuroticism score, depressive symptoms, age group, ethnicity, education level, region, long-standing illness, socioeconomic deprivation and years between baseline and follow-up
Model 2: Model 1 + smoking + alcohol consumption + BMI

References

1. UK Biobank. Repeat Assessment Data. Available at: https://biobank.ctsu.ox.ac.uk/~bbdatan/Repeat_assessment_doc_v1.0.pdf. 2013.
2. UK Biobank. Repeat Assessment: Participant Characteristics of responders vs. non-responders. Available at: http://biobank.ctsu.ox.ac.uk/~bbdatan/repeat_assessment_characteristics_v1.pdf. 2014.
3. Martin A, Rief W, Klaiberg A, Braehler E. Validity of the Brief Patient Health Questionnaire Mood Scale (PHQ-9) in the general population. *General Hospital Psychiatry.* 2006;28(1):71-77.
4. Kroenke K, Spitzer RL, Williams JB, Löwe B. An ultra-brief screening scale for anxiety and depression: the PHQ–4. *Psychosomatics.* 2009;50(6):613-621.
5. Lyall LM, Wyse CA, Celis-Morales CA, et al. Seasonality of depressive symptoms in women but not in men: A cross-sectional study in the UK Biobank cohort. *Journal of Affective Disorders.* 2018;229:296-305.
6. Townsend P. Deprivation. *Journal of social policy.* 1987;16(2):125-146.
